# Microporous N-Doped Carbon Obtained from Salt Melt Pyrolysis of Chitosan toward Supercapacitor and Oxygen Reduction Catalysts

**DOI:** 10.3390/nano12071162

**Published:** 2022-03-31

**Authors:** Maria Krystyna Rybarczyk, Karolina Cysewska, Recep Yuksel, Marek Lieder

**Affiliations:** 1 Chemical Faculty, Warsaw University of Technology, Noakowskiego 3, 00-664 Warsaw, Poland; 2 Faculty of Electronics, Telecommunications and Informatics, Gdansk University of Technology, Narutowicza 11/12, 80-233 Gdansk, Poland; karolina.cysewska@pg.edu.pl; 3 Department of Chemistry, Faculty of Science and Letters, Eskisehir Osmangazi University, 26040 Eskişehir, Turkey; recep.yuksel@ogu.edu.tr; 4 Department of Process Engineering and Chemical Technology, Chemical Faculty, Gdansk University of Technology, Narutowicza 11/12, 80-233 Gdansk, Poland; lieder@pg.edu.pl

**Keywords:** carbon nanostructure, electrocatalysts, supercapacitors, N-rich nano-porous carbon

## Abstract

The direct carbonization of low-cost and abundant chitosan biopolymer in the presence of salt eutectics leads to highly microporous, N-doped nanostructures. The microporous structure is easily manufactured using eutectic mixture (ZnCl_2_-KCl) and chitosan. Potassium ions here can act as an intercalating agent, leading to the formation of lamellar carbon sheets, whereas zinc chloride generates significant porosity. Here, we present an efficient synthetic way for microporous carbon nanostructures production with a total nitrogen content of 8.7%. Preliminary studies were performed to show the possibility of the use of such material as a catalyst for supercapacitor and ORR. The textural properties enhanced capacitance, which stem from improved accessibility of previously blocked or inactive pores in the carbon structure, leading to the conclusion that porogen salts and molten salt strategies produce materials with tailor-made morphologies. The synergistic effect of the eutectic salt is seen in controlled porous structures and pore size, and the micropores boosting adsorption ability.

## 1. Introduction

Biomass, as the most abundant renewable resource, is currently intensively used for biofuels/chemicals production or can serve as a precursor for the production of porous carbon materials [1,2,3,4,5,6,7]. Chitosan (CS) is an *N*-deacetylated product of chitin, which is easily soluble in solvents capable of disrupting hydrogen bonds, e.g., dilute aqueous solutions of organic and mineral acids or some ionic liquids. The production of chitosan-based activated carbons is led by employing a solution- and/or solid-phase synthesis pathway [8,9,10]. Furthermore, a simple heat-treatment of chitosan in an inert gas atmosphere results in random carbons with no specific porosity (S_BET_ < 10 m^2^) [8,11]. However, the controlled synthesis of chitosan-based porous carbon materials has also been studied through template or activation methods. Specifically, the application of templates such as Zn salts [12,13] Na/K carbonate [8,9], silica [14], or boric acid [15]; and activators like KOH [16,17] have resulted in N-doped nanocarbon materials with highly microporous structures possessing gas sorption capability, and electrochemical or electrocatalytic activity [18,19].

A promising alternative to the aqueous- or solid-phase synthesis of high-surface-area and aerogel-like (highly porous) nanocarbons are provided by ionic melts (IM) as reaction media. These solvents are reported to possess many advantages, including low cost, nontoxicity, wide operational temperatures, and recyclability [20]. Another significant advantage of IM is their potential for the controlled synthesis of highly anisotropic materials [21,22]. Morphology control, particularly for catalytic materials, is of great importance, since their activity at the nanoscale is strongly influenced by shape and dimension [23]. Salt templating is a process during which a non-carbonizable inorganic salt is mixed with a carbon precursor. It is claimed that the miscibility between the salt melt and the carbonizing material should be retained over the main part of the reaction pathway [24]. Nevertheless, the crucial parameter during the salt-templating is the percolation mechanism [25]. The molten salts offer the possibility for an active reaction media, provided the carbonaceous precursors are soluble in it and if a space-oriented interaction of carbonised intermediates with the salt ions takes place. For example, zinc chloride (ZnCl_2_) can act as a dehydration agent for cellulosic materials and therefore promotes the formation of reactive double bonds, cyclo-additions, and the formation of carbon skeleton [26,27,28]. It was proven that biomass could serve as a sustainable and renewable source for different carbon structures, including graphene. A facile molten-salt route to graphene synthesis with soluble glucose was reported by Antonietti’s group [29]. The ZnCl_2_ based melts were also reported to actively drive carbonization of ionic liquids with glucose, polysaccharides, or biomass [30,31] into specific morphology, resulting in high surface area carbons. So far, hierarchical porous carbons have been obtained by the templating approach with silica spheres of a diameter of 14 nm from pure chitosan or a chitosan/ZnCl_2_ solution resulting in the creation of microporosity [32].

Individual potassium chloride (KCl) has high heat capacities (0.99 kJ kg^−1^ K^−1^), low vapor pressure at high temperatures, and weak hygroscopy, but high melting temperatures (771 °C) [30]. Moreover, higher amounts of KCl in the mixture can shield the Lewis acidity of ZnCl_2_, thereby lowering their miscibility [33]. Here, other parameters such as polarity should also be addressed. Apart from the fact that both KCl and ZnCl_2_ act as dehydrating agents, they demonstrate the synergistic role of the components and their derivatives, such as K_2_ZnCl_4_ with an intermediate phase that exists within a narrow temperature range between 110 and 130 °C [34]. The use of 800 °C further explains the dehydration, preliminary deacetylation, and main chitosan chain degradation up to 300 °C [35].

The biomass-derived porous carbon structures are suitable electrode active materials for electrochemical energy storage systems due to their high surface area, microporous structure, good electrochemical stability, and good electronic and ionic conductivities. Electrostatic charges accumulate on the accessible pores and the active surface area of the carbon materials in the supercapacitor electrode. While the micropores of the carbon structures are advantageous for energy density, the interconnected pores and larger pores are good for power densities. Thus, electrochemical energy storage systems, particularly supercapacitors, require micro- and interconnected porous carbon materials as electrode materials to achieve high performance [36,37,38].

Here, we demonstrate the successful synthesis of carbon materials, where chitosan serves as a carbon precursor and ZnCl_2_/KCl as a pore padding agent and solvent at elevated temperature. A relatively high ZnCl_2_/KCl-to-chitosan weight ratio was adopted to ensure that the salt melt is retained over the main part of the carbonization pathway in order to facilitate the formation of microporous carbon structures. In our previous report, we focused on the formation of curved structures in the grown graphitic layers which stem from the presence of Li [35]. We chose the ZnCl_2_/KCl eutectic mixture since its melting point lies within the temperature range of chitosan decomposition while the salts remaining, after the carbonization, can be washed out with water or dilute inorganic acids. The results indeed indicate that the use of chitosan as a precursor can lead to the onset of carbon solidification in a complete liquid reaction mixture, pointing to the desired salt melt process as a carbonization mechanism showing the high compatibility of the used chitosan with the ionothermal approach. The as-obtained, functionalised nitrogen-doped carbon nanostructures were used as electrocatalysts for oxygen reduction, exhibiting a high reaction onset potential in alkaline electrolytes and also showing electrochemical charge storage activity as a supercapacitor electrode active material.

## 2. Materials and Methods

### 2.1. Materials

Chemical reagents were purchased through commercial vendors:

- Chitosan, medium molecular weight (M, 210–300 kDa) and deacetylation degree 84% (Sigma-Aldrich, Darmstadt, Germany)
- Anhydrous Potassium chloride (Sigma-Aldrich, Darmstadt, Germany)
- Anhydrous Zinc chloride (Sigma-Aldrich, Darmstadt, Germany).

All reagents were used without further purification.

### 2.2. Methods

In order to fabricate N-carbon, chitosan (3 g) was mixed with a eutectic salt (4.5 g) mixture consisting of 49 mol% ZnCl_2_ + 51 mol% KCl and carbonised at 800 °C under N_2_ atmosphere. In order to remove any inorganic salt residuals from the carbonaceous species, the carbonized powder was agitated thoroughly by sonication in 3.5 M HCl and filtrated until the solution became neutral. The final form of carbon powder (CH_K) was dried in a vacuum dryer for 24 h at 80 °C and stored in a desiccator storage. The presented conditions and parameters for the fabrication of N-doped carbons were chosen during the optimization procedure in order to obtain the most efficient catalytic activity of the material.

### 2.3. Analysis and Characterization Techniques

The morphology of the samples was analysed by scanning electron microscopy (SEM) FEI Quanta 3D ESEM/FIB (SEM, Hillsboro, OR, USA) equipped with an SE detector (10 kV beam accelerating voltage). The nitrogen-containing functional groups present on the carbon surface were determined by X-ray photoelectron spectroscopy (XPS) using an VG Scienta250Xi spectrometer (XPS, PREVAC Sp. z o. o. Rogow, Poland) with Al Ka radiation (hv = 1486.6 eV). All samples were measured at room temperature under ultra-high vacuum with pressures below 1.1 × 10^−8^ mBar. The binding energy scale was regulated by setting the C 1s transition at 284.6 eV. The Raman spectra were recorded using the Renishaw 2000 system with excitation by diode laser (514 nm, laser power of 25 mW) in backscattering geometry. The XRD patterns were recorded by a Siemens D5000 X-Ray Powder diffractometer (XRD, Siemens Diffraktometer-D5000, Siemens, Berlin, Germany) with Cu radiation. Each pattern was recorded with a step size of 0.03°. The pore size distribution and surface area were analysed by the quenched solid density functional theory (QSDFT) and Brunauer–Emmett–Teller (BET) method, respectively. The total surface area was measured by Quantachrome NovaWin Version 11.03 (Quantachrome Instruments, Boynton Beach, FL, USA). The elemental analysis was carried out on the CHN Analyser 2400 (Perkin-Elmer, Rodgau, Germany) by a conventional CHN combustion method.

The electrochemical measurements for oxygen reduction reaction (ORR) were carried out in an aqueous solution of 0.1 M KOH in a 3-electrode system controlled by Autolab PGSTAT 101. A glassy carbon (GC) rotating electrode (diameter = 3 mm) with N-carbon powder coating was used as a working electrode. A platinum wire and an Ag/AgCl electrode filled with saturated KCl aqueous solution were used as a counter and reference electrode, respectively. The catalyst ink was prepared by blending the catalyst powder (1.5 mg) with 50 µL of isopropanol, 50 µL of Nafion, and 900 µL of DI water and sonicated for 30 min. Then, a 5 µL droplet was put on the surface of the working electrode and it was dried for 30 min under air atmosphere. Cyclic voltammetry (CV) measurement was performed from 0.2 V to −1.2 V with a scan rate of 50 mV s^−1^. Linear scan voltammetry (LSV) measurement was carried out from 0.2 V to −1.2 V with a scan rate of 10 mV s^−1^ (400, 800, 1200, 1600, 2000, and 2400 rpm). All the potentials in the work (for electrocatalyst ORR measurements) were converted into reversible hydrogen electrode (RHE) scale by adding 0.965 V. The background current in ORR was subtracted from the capacitive current by conducting LSV measurements in both N_2_ and O_2_, and then by removing the current of N_2_ from that of O_2_ in order to get rid of the significant double-layer capacitance for carbonaceous materials.

The electrodes for the application of supercapacitors were prepared using N-doped carbon nanomaterial, conductive additive carbon black, and binder polytetrafluoroethylene (PTFE) at a weight ratio of 8:1:1, respectively. Fabricated circular electrode discs were cut into 8 mm diameter and dried in a vacuum oven at 80 °C for 3 h. Electrochemical measurements were conducted in a two-electrode configuration using a Zivelab MP1 potentiostat/galvanostat system (Zivelab, Seoul, South Korea) Lab-build Swagelok cells were used for the electrochemical measurements. The mass of the active material in the fabricated identical electrodes for the symmetrical supercapacitor device was measured as 9.45 mg/electrode. The areal active material loading was 18.9 mg/cm^2^.

## 3. Results

**Figure 1:**
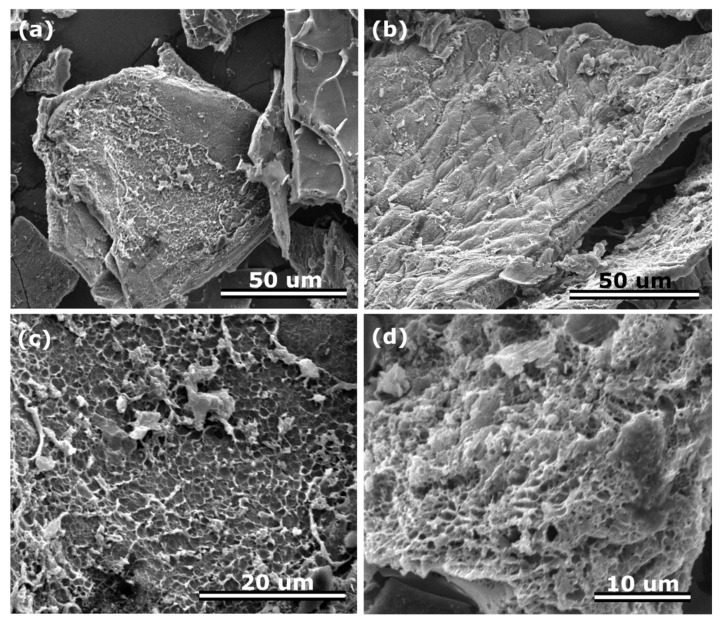
SEM images (**a**–**d**) of CH_K with different magnifications.

The morphology of the CH_K materials was studied using scanning electron microscopy (Figure 1).

The SEM images clearly shows an open structure of well-separated lamellar carbon particles with pores in the framework. Moreover, the structure in some parts is characterized with an interconnected 3D porous network. The latter is highly desirable, especially in the case of electrocatalysis, i.e., it might support fast transport of ions due to the easily accessible open spaces. On the other hand, in contrary to the physical one, the as-used chemical activation can tailor microporosity [39].

**Figure 2:**
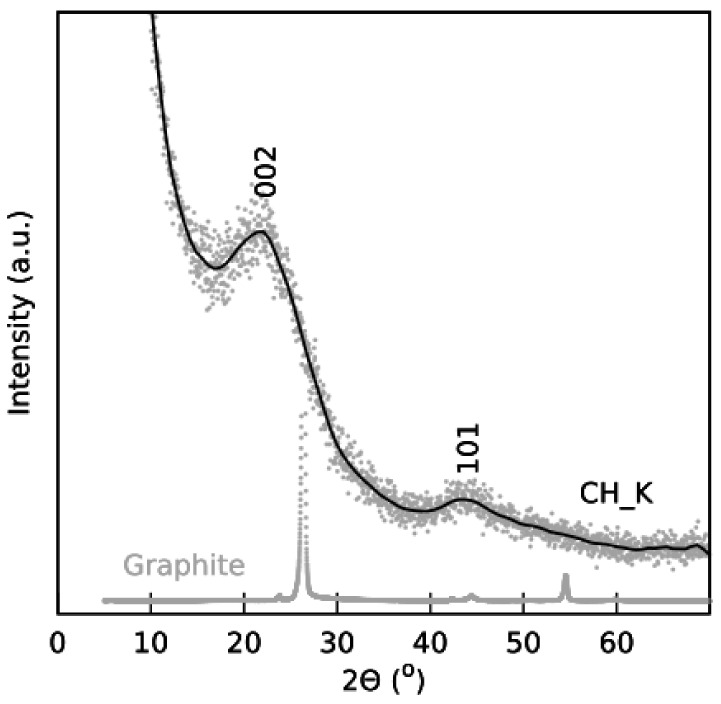
XRD patterns of CH_K.

The structure of the CH_K samples was analyzed by X-ray diffraction (XRD) (Figure 2).

The XRD peak at 22° describes carbon material with d_002_ spacing of 3.440 Å (graphitized carbon) while the second XRD hump with corresponding d_101_ spacing implies a combination of many turbostratic carbonaceous domains parallel to the graphitized carbon.

**Figure 3:**
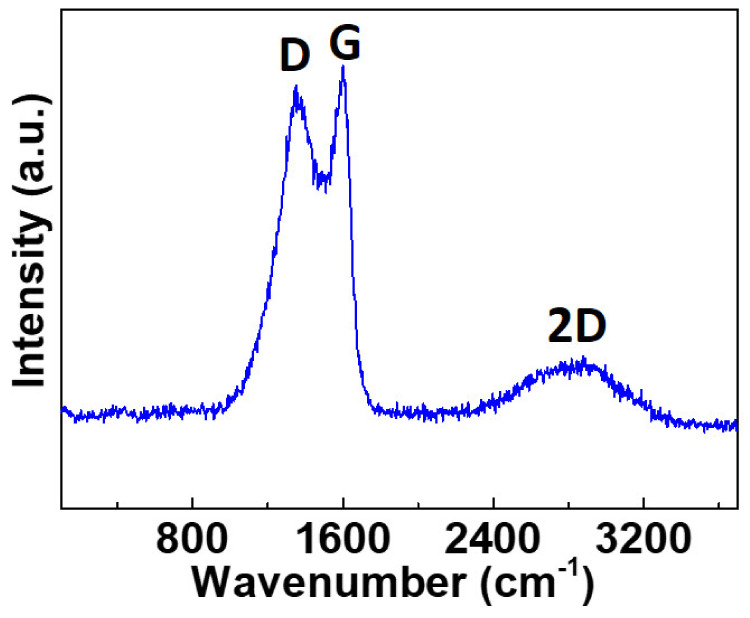
Raman spectra of the N-doped CH_K carbons.

Raman spectroscopy is an important tool to investigate the structural properties of carbon-based materials. Figure 3 shows three distinct peaks of the fabricated CH_K materials observed at 1348, 1599, and 2876 cm^−1,^ which belong to the D, G, and 2D bands, respectively [35]. The intensity ratio (I_D_/I_G_) of D and G bands is 0.98, and this ratio may reflect a defective/porous structure for the carbon material. The 2D band is the second order of the two-phonon process and can be used to determine the number of graphene layers. The observed D band shows that the fabricated carbon material is formed from a few carbon layers.

**Figure 4:**
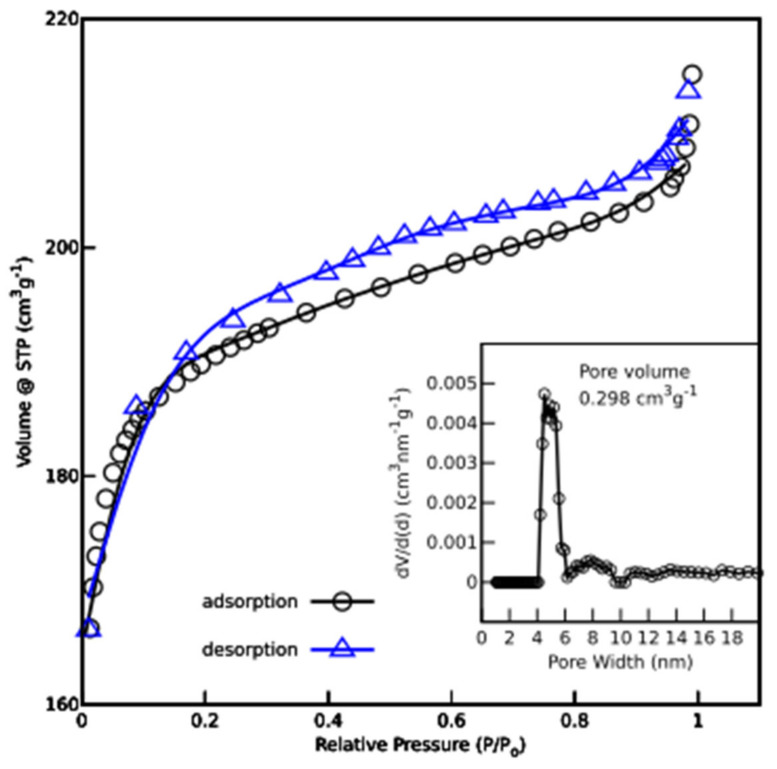
Nitrogen isothermal sorption with their corresponding pore size distribution (inset) of CH_K.

The BET surface area reached up to 606.42 m^2^ g^−1^, revealing a huge micropore contribution with a pore width (Mode) of 1.007 nm to the total surface exposure. Major adsorption phenomena occur at a low relative pressure of less than 0.2, indicating high microporosity in the sample (Figure 4). The detailed pore fractions are listed in Table 1. The reversible type I isotherm presented here is supposed to have a relatively small external surface since the concave to the P/P_0_ axis is limited. Here, it needs to be underlined that micropores result from the direct carbonization, enhanced by the environment of the conducted pyrolysis. On the contrary, the simple carbonization displays no porosity with a low specific surface area.

**Figure 5:**
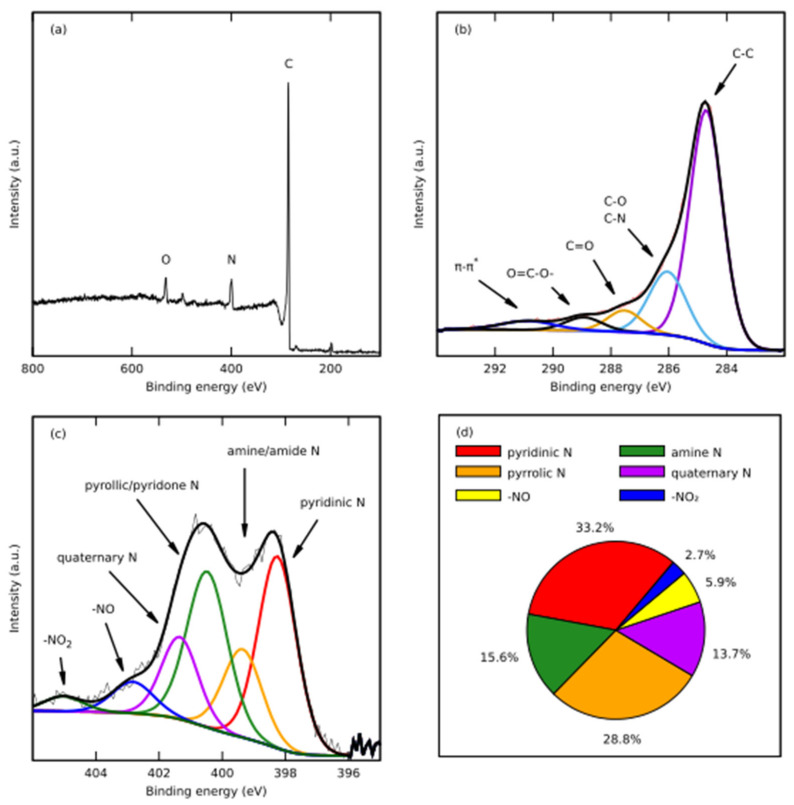
(**a**) XPS spectra of CH_K carbons; high-resolution (**b**) C 1s and (**c**) N 1s of N-doped carbon; (**d**) atomic percentage of the nitrogen functional groups in CH_K.

The chemical composition of CH_K was investigated by X-ray photoemission spectroscopy (XPS) (Figure 5). Three distinct peaks at around 532 eV, 400 eV, and 285 eV, corresponding to O 1s, N 1s, and C 1s, respectively, were observed in the XPS survey spectra (Figure 5a). The high-resolution C 1s spectrum can be additionally deconvoluted into five peaks corresponding to C–C (284.7 eV), C–O/C–N (286.0 eV), C=O (287.5 eV), O=C–O (288.9 eV), and π-π* (290.7 eV) (Figure 5b). Deconvolution of the high-resolution N 1s spectrum into six specific peaks was also performed (Figure 5c). The peaks can be attributed to the pyridinic N (398.3 eV), amine/amide N (399.4 eV), pyrollic/pyridone N (400.7 eV), and quaternary N (401.5 eV); and the latter ones to –NO and –NO_2_ functional groups. The pyrollic and pyridone N constitute the highest atomic percentage i.e., 33.2 and 28.8%, respectively, in the material (Figure 5d). The XPS analysis confirms the successful doping of N into the carbon material. It was reported that the presence of the pyrollic and pyridone N at the edge of the carbon skeleton significantly increases the capacitive performance of the material [40].

In order to investigate the elemental composition in the bulk of CH_K catalysts, the CHN method was used. The results demonstrate successful nitrogen doping up to 8.36 %mas. in the bulk and up to 8.7%mas. on the surface (Table 2).

**Figure 6:**
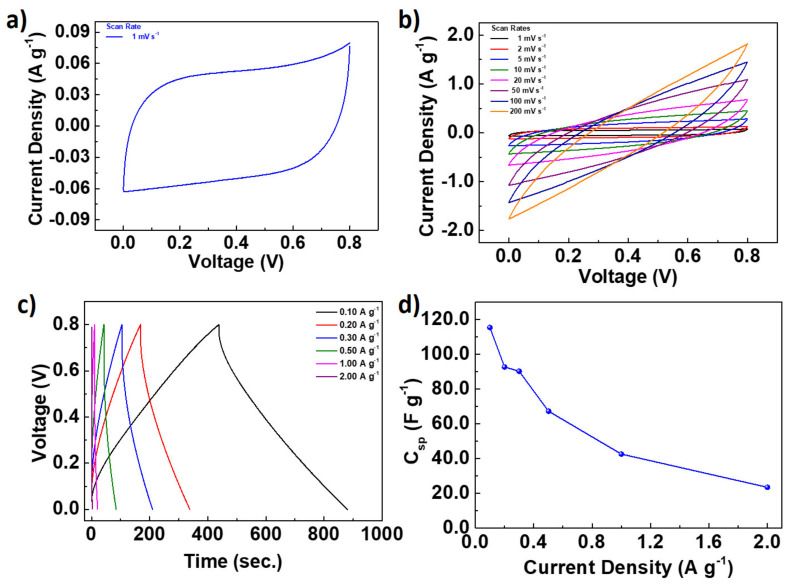
(**a**) CV results of the fabricated CH_K supercapacitors at a scan rate of 1 mV s^−1^. (**b**) CV results of the fabricated devices at different scan rates. (**c**) GCD results of the fabricated supercapacitors at different current densities. (**d**) C_sp_ of the CH_K supercapacitors as a function of current density. Lines are for visual aid.

The electrochemical energy storage properties of the fabricated CH_K are investigated using symmetrical supercapacitor devices with 1.0 M H_2_SO_4_ aqueous electrolyte in a voltage window of 0–0.8 V. CV measurement of the fabricated device was performed at a scan rate of 1 mV s^−1^; the obtained CV result is provided in Figure 6a. The obtained CV curve has an ideal shape that reflects an electric double-layer capacitor (EDLC)-type behavior. As shown in Figure 6b, the CV measurements are also repeated at high scan rates. The fabricated supercapacitor also shows encouraging electrochemical performance at high scan rates and almost preserves its rectangular shape. During the CV measurements, the slight deviations from the rectangular shape of the CV curves at high scan rates may be because of the resistivity, which is created by the limited accessibility of inner pores during the charge transfers. The electrolyte ions inside the narrow inner pores of the carbon materials may not respond fast enough at high current densities during the charge polarization. The slow or delayed interaction of the electrolyte ions may increase the resistivity, which results in deviation from the rectangular curve shape. The obtained results reflect that the fabricated supercapacitors have good charge transfer and storage performance. The charge storage properties of the fabricated supercapacitor device were also examined by galvanostatic charge-discharge (GCD) measurements at various current densities, the results of which are provided in Figure 6c. The fabricated device had almost linear charge–discharge profiles and showed similar charge–discharge profiles at different current densities with a limited internal resistance drop (IR_drop_). At 0.1 A g^−1^ current density, the device was fully charged in 439 **s** and then discharged at the same time, which corresponds to 100% Coulombic efficiency. High Coulombic efficiency is also preserved at high current densities for GCD measurements. The specific capacitance (C_sp_) of the N-doped carbon nanomaterial at various current densities were calculated from the GCD curves (Figure 6c); the results are plotted in Figure 6d. C_sp_ was calculated as 115.4 F g^−1^ at a current density of 0.10 A g^−1^ for the fabricated symmetrical supercapacitor device. When the current density was increased to 1.0 A g^−1^, C_sp_ was found as 42.5 F g^−1^. The fabricated device demonstrated a reasonable rate capability (36.8%); even the current density increased ten-fold. The obtained results show that the N-doped carbon nanomaterial has high energy storage and rate capability properties.

**Figure 7:**
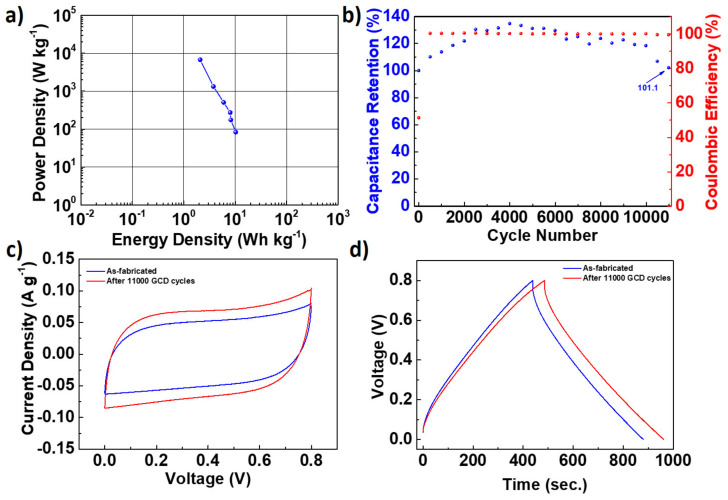
(**a**) Ragone plot of the fabricated supercapacitor. (**b**) The capacity retention of the fabricated supercapacitor was measured with a constant GCD at 1.0 A g^−1^ over a voltage range of 0–0.8 V. Comparison of (**c**) CV and (**d**) GCD of as-fabricated and supercapacitors tested over 11,000 cycles.

A Ragone plot was drawn to compare the energy and power densities of the fabricated supercapacitor and is shown in Figure 7a. The energy density of the supercapacitor was found to be between 2.08 and 10.3 Wh kg^−1^ at a power density range of 83.6–6772.5 W kg^−1^. These energy densities of the N-doped carbon nanomaterial are quite reasonable when considered as carbon-based nanomaterials. A long-term GCD test is an important parameter to evaluate a supercapacitor’s cycle life and capacity retention. Figure 7b provides the cycle life results for the fabricated supercapacitor at a current density of 1 A g^−1^. The supercapacitor showed high durability and its capacitance retention was found to be 101.1% after 11,000 GCD cycles. The high capacitance retention may result from the highly porous and interconnected nature of the CH_K nanomaterial. The CV and GCD measurements were repeated after 11,000 GCD cycles; the obtained results are provided in Figure 7c,d, respectively. Both results show that the charge storage performance of the fabricated devices is improved. The C_sp_ was calculated as 125.1 F g^−1^ at 0.10 A g^−1^ after 11,000 GCD cycles, corresponding to an 8.4% capacitance increase. The enhanced capacitance can be due to the accessibility of previously blocked or inactive pores in the carbon structure. The activated pores provide rapid electron and ion transfer and a high charge accumulation on the electrode surface. The obtained results are highly encouraging for the N-doped carbon nanomaterial, which seems to be promising electrode material for high-performance, carbon-based energy storage applications.

**Figure 8:**
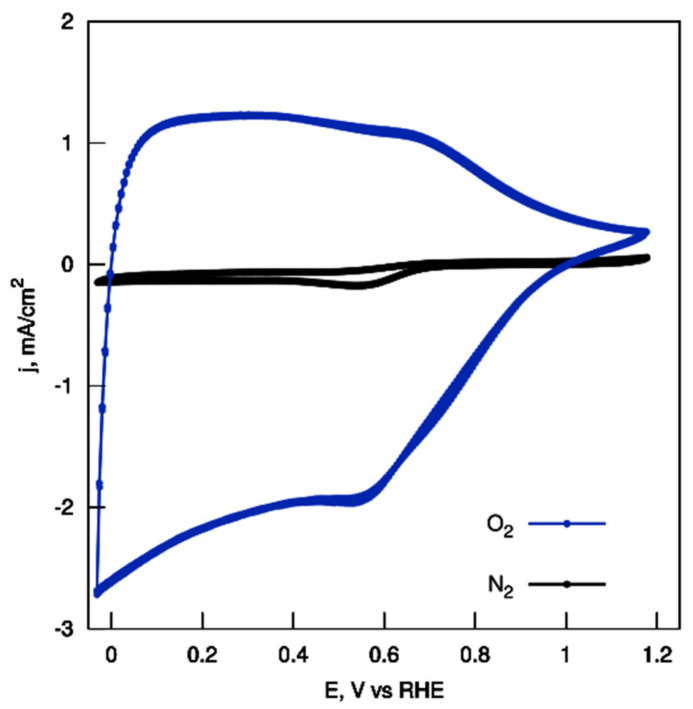
CV measurements at a scan rate of 10 mV s^−1^ for CH_K.

The ORR catalytic activity of the CH_K material was initially investigated using CV measurements in O_2_ or N_2_ saturated 0.1 M KOH at a scan rate of 10 mV/s (Figure 8). A voltammogram with only a minute cathodic peak was obtained for the catalyst in the absence of oxygen. After saturating the solution with oxygen, a wave of the reduction current of ca. 2 mA/cm^2^ can be seen at potentials lower than 0.55 V.

**Figure 9:**
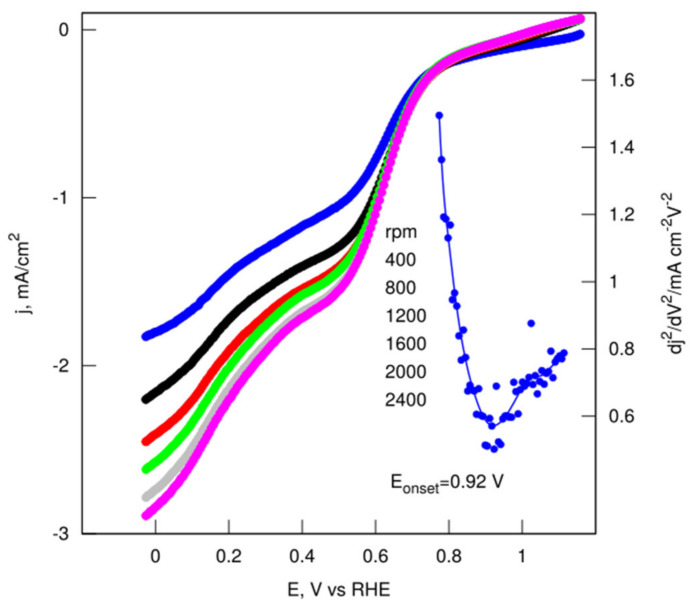
LSV measurements at a scan rate of 10 mV s^−1^ for CH_K.

To further investigate the ORR activity parameters, LSV measurements were performed on a rotating disk electrode (RDE) at rotations from 400 to 2400 rpm in O_2_-saturated 0.1 M KOH. The ORR onset potentials of the sample were acquired from an RDE linear sweep at 1600 rpm (Figure 9). Remarkably, the highly-porous CS showed obviously good performance, both in terms of the onset potential (ca. 0.92 V vs. RHE) and the reaction current density.

**Figure 10:**
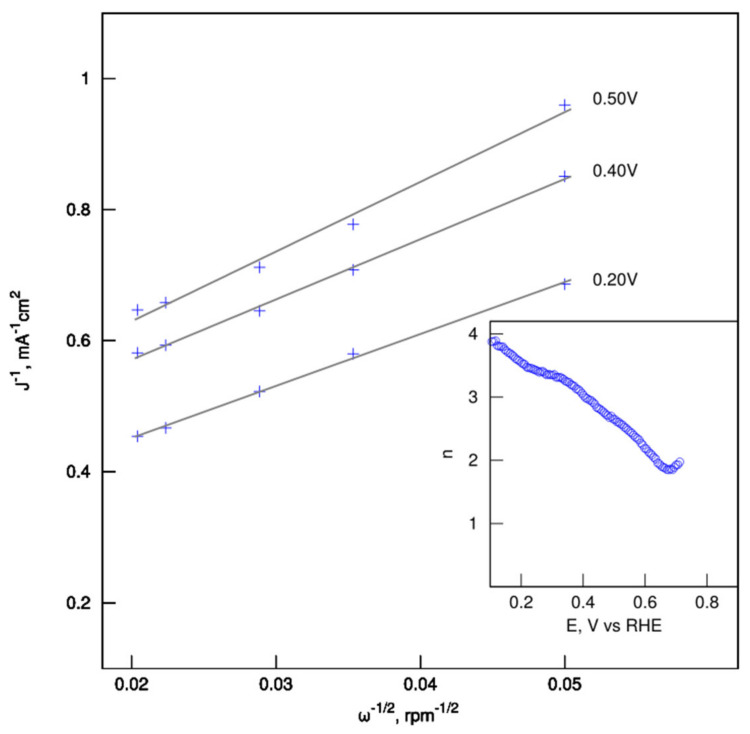
The Koutecky–Levich plots and moles of electrons involved in the ORR for CH_K catalysts.

The Koutecky–Levich plots (J^−1^ vs. ω^−1/2^) of the catalyst were obtained from LSVs according to the current at various potentials and all lines show good linearity (Figure 10). The electron-transfer number calculated from the slope of Koutecki–Levich plots for the electrode potential in the range from 0.1 to 0.6 V is not constant and decreases from 4 at low overpotential to ca. 2 at 0.6 V.

The changing electron transfer number implies that the catalytic sites in the material vary in terms of adsorption affinity of oxygen onto them [35].

At low overpotential, those that catalyse 4-electron reduction of oxygen to water are more active: O_2_ + 2H_2_O + 4e^−^ = 4OH^−^; whereas at higher voltages, reduction of oxygen to H_2_O_2_ prevails 2e^−^ route: O_2_ + H_2_O + 2e^−^ = HO_2_^−^ + OH^−^. The change in reaction pathway induced by a driving force is nothing new in electrochemistry; however, in this case, the reasons for the observed phenomenon are not clear to the authors.

## 4. Discussion and Conclusions

The direct carbonization of low-cost and abundant chitosan biopolymer in the presence of salt eutectics leads to highly microporous nanostructures. The molten ZnCl_2_ salt offers the possibility for an active reaction media, provided the carbonaceous precursors are soluble in it and if a space-oriented interaction of carbonized intermediates with the salt ions takes place.

The as-reported synthesis protocol leads to carbon materials with BET surface area up to 606.42 m^2^ g^−1^, revealing a huge micropore contribution to the total surface exposure. The textural properties enhanced capacitance, which stem from improved accessibility of previously blocked or inactive pores in the carbon structure. The nitrogen-doping level reached up to 8.36% mas. in bulk and up to 8.7% mas. on the surface, which, in parallel to oxygen functionality and porosity, boosts the adsorption ability for electrocatalytic reactions [41,42]. We assume pyridinic or/and pyrrolic groups catalyse oxygen reduction by facilitating oxygen molecules’ adsorption, followed by their splitting and charge transfer. Other N species embedded in the carbonaceous structure (CH_K), such as –NH_2_, have little effect on the electrochemical performance of the catalyst. The obtained results reflect that the fabricated supercapacitors have good charge transfer and storage performance.

Indeed, the pore formation mechanism envisages the ion pairs/salt clusters and their percolation [43], leading to the conclusion that the porogen salts and molten salt strategies produce materials with tailor-made morphologies. The preparation of porosity with ZnCl_2_ alone during impregnation affects the surface area and the micropore size distribution [44], while in our report, the use of the salt melt (49 mol% ZnCl_2_+ 51 mol% KCl) is crucial for the microporosity framework. On the other hand, the use of chitosan has a positive effect on capacity since the chitosan-based binary metal oxide material for energy storage devices is considered environmental-friendly. Ramkumar and Minakshi have indicated that chitosan gel strongly adheres to the molybdate moiety of CoMoO_4_ and increases the capacitance (81 F g^−1^) four-fold, compared to a chitosan-free material (17 F g^−1^) [45]. Importantly, other waste material can serve as sustainable electrode materials. It has been reported that calcined eggshells (CaO) electrodes exhibit 55 F g^−1^ at a current density of 0.15 A g−1 with a capacitance retention of nearly 100% after 1000 GCD cycles. In another report from the same group, calcined biomass-derived electrodes also showed similar capacitance and retention values. Unlike the above references, the precursor material (chitosan) in this work yielded a carbon-based material and the obtained specific capacitance value was 115.4 F g^−1^ at a current density of 0.10 A g^−1^ [46,47].

In summary, we demonstrated a simple synthetic route to prepare carbon clusters and microporous voids from chitosan pyrolyzed in a molten eutectic salt mixture of ZnCl_2_ and KCl. These microstructures were formed by a direct percolation mechanism induced by the molten salts’ ‘mould’. The incorporation of nitrogen and oxygen heteroatoms into the carbonaceous microporous structure can change charge localization and positively charged species for electrocatalytic reactions influencing chemisorption. This widens the applications of emerging technologies for energy storage and conversion circular economy in the design and development of power cells.

## Figures and Tables

**Table 1:** Summary of BET surface area and pore volumes.

Name	Total Area m^2^ g^−1^	Micro-Pore Area m^2^ g^−1^	Meso-Pore Area m^2^ g^−1^	Total Pore Volume cm^3^ g^−1^	Micropore Volume cm^3^ g^−1^	Mesopore Volume cm^3^ g^−1^	Pore with nm
CH800 ^a^	10.5	n/a	n/a	0.03	n/a	n/a	3.939
CH_K	606.42	553	6.775	0.298	0.285	0.013	1.007

^a^ Chitosan carbonised at 800 °C.

**Table 2:** Elemental analysis using the CHN combustion method and XPS data.

	Precursor	CH_K	
		[%mas.] CHN	XPS
C	38.3	80.0	83.3
H	6.7	2.02	-
N	5.9	8.36	8.7
O	49.1 ^b^	9.62 ^b^	8.0

^b^ Calculated as the difference.

## Data Availability

The data presented in this study are available on request from the corresponding author.
